# Phase II study of multidisciplinary therapy combined with pembrolizumab for patients with synchronous oligometastatic non-small cell lung cancer TRAP OLIGO study (WJOG11118L)

**DOI:** 10.1186/s12885-021-08851-z

**Published:** 2021-10-18

**Authors:** Taichi Miyawaki, Hirotsugu Kenmotsu, Hideyuki Harada, Yasuhisa Ohde, Yasutaka Chiba, Koji Haratani, Tamio Okimoto, Tomohiro Sakamoto, Kazushige Wakuda, Kentaro Ito, Takehiro Uemura, Shinya Sakata, Yoshihito Kogure, Yasumasa Nishimura, Kazuhiko Nakagawa, Nobuyuki Yamamoto

**Affiliations:** 1 Division of Thoracic Oncology, Shizuoka Cancer Center, 1007 Shimonagakubo, Nagaizumi-cho, Sunto-gun, Shizuoka, 411-8777 Japan; 2 Department of Respiratory Medicine, Juntendo University Graduate School of Medicine, Tokyo, Japan; 3 Radiation and Proton Therapy Center, Shizuoka Cancer Center, Shizuoka, Japan; 4 Division of Thoracic Surgery, Shizuoka Cancer Center, Shizuoka, Japan; 5 Clinical Research Center, Kindai University Hospital, Osaka-sayama, Japan; 6 Department of Medical Oncology, Kindai University Faculty of Medicine, Osaka-sayama, Japan; 7 Department of Internal Medicine, Division of Medical Oncology & Respiratory Medicine, Shimane University Faculty of Medicine, Izumo, Japan; 8 Division of Respiratory Medicine and Rheumatology, Department of Multidisciplinary Internal Medicine, Tottori University, Yonago city, Japan; 9 Respiratory Centor, Matsusaka Municipal Hospital, Matsusaka, Japan; 10 Department of Respiratory Medicine, Allergy and Clinical Immunology, Nagoya City University Graduate School of Medical Sciences, Nagoya, Japan; 11 Department of Respiratory Medicine, Kumamoto University Hospital, Kumamoto, Japan; 12 Department of Respiratory Medicine, National Hospital Organization Nagoya Medical Center, Nagoya, Japan; 13 Department of Radiation Oncology, Kindai University Faculty of Medicine, Osaka-sayama, Japan; 14 Internal Medicine III, Wakayama Medical University, Wakayama, Japan

**Keywords:** Clinical trial, Programmed cell death 1 inhibitor, Oligometastatic disease, Local ablative therapy, Progression-free survival

## Abstract

**Background:**

Synchronous oligometastatic non-small cell lung cancer (NSCLC) is generally characterised by the limited number of metastases at the time of diagnosis. Several clinical trials have shown that local ablative therapy (LAT) at all sites of the disease might be beneficial for patients with oligometastatic NSCLC. In recent years, the combination of programmed cell death 1 (PD-1) inhibitors or programmed cell death ligand 1 with cytotoxic chemotherapy has become a new standard treatment for patients with metastatic NSCLC. Furthermore, multisite LAT would inherently reduce the overall tumour burden, and this could promote T cell reinvigoration to enhance the efficacy of PD-1 inhibitors. Few studies have evaluated the efficacy of the combination of PD-1 inhibitors with LAT at all sites of disease. The aim of the present multicentre single-arm phase II study is to evaluate the efficacy of LAT at all sites of disease following standard platinum doublet chemotherapy with pembrolizumab in patients with oligometastatic NSCLC.

**Methods:**

Thirty patients with synchronous oligometastatic NSCLC will be enrolled in the trial. All patients will receive 2–4 cycles of a systemic treatment including pembrolizumab and chemotherapy as induction therapy. Patients who will receive LAT will be determined by a multidisciplinary tumour board, including medical oncologists, radiation oncologists, and thoracic surgeons. LAT will be administered at all sites of disease within 21–56 days of the last dose of induction therapy and will be followed by maintenance therapy within 42 days of the last day of LAT. The primary endpoint is the progression-free survival (PFS) rate of 24 months from the date of initiation of LAT. The secondary endpoints are toxicity, response to induction therapy, PFS, overall survival, and the frequency of LAT.

**Discussion:**

This study will provide novel data on the efficacy and safety profile of the combination of LAT and chemotherapy plus immune-checkpoint inhibitors in patients with synchronous oligometastatic NSCLC. If the primary endpoint of this study is met, extensive phase III studies further assessing this strategy will be recommended.

**Trial registration:**

jRCT identifier: jRCTs041200046 (date of initial registration: 28 October 2020).

## Background

Synchronous oligometastatic non-small cell lung cancer (NSCLC) is generally characterised by the limited number of metastases at the time of diagnosis [1]. In recent years, oligometastatic NSCLC has been defined as a disease stage harbouring few metastases treatable with local ablative therapies (LAT). Predominant patterns of initial progressive disease (PD) after first-line systemic therapy suggested the utility of LAT for all sites in patients with oligometastatic NSCLC. Several prospective trials for patients with oligometastatic NSCLC suggested that LAT might be beneficial [2, 3]. Furthermore, a randomised phase II trial in patients with oligometastatic NSCLC (1–3 metastases) has shown that LAT at all sites of the disease is associated with a significant improvement in overall survival (OS) and progression-free survival (PFS) compared with maintenance therapy alone [4, 5]. Another randomised phase II trial in oligometastatic NSCLC patients with 1–5 metastases also showed that LAT to all sites of disease after systemic therapy is associated with a significant improvement in PFS compared with maintenance therapy alone [6]. In both trials, cytotoxic chemotherapy or targeted therapy could only be administered as systemic therapy. In recent years, programmed cell death 1 (PD-1) inhibitors have revolutionised the treatment of NSCLC. The KEYNOTE-189 and KEYNOTE-407 studies showed that the addition of pembrolizumab to platinum doublet chemotherapy improved OS in patients with non-squamous and squamous NSCLC, regardless of programmed cell death ligand 1 expression [7, 8].

Some studies suggested that tumour burden was correlated with exhausted CD8 T-cells, which is a negative prognostic indicator for anti-PD-1 inhibitors [9, 10]. Furthermore, pre-clinical studies suggested that tumour debulking improved treatment outcomes [11]. LAT to all sites of disease that reduce tumour burden as much as possible might maximise synergy effects with PD-1 inhibitors in patients with oligometastatic NSCLC. In fact, the phase II trial of pembrolizumab after LAT for oligometastatic NSCLC showed promising results with a two-year OS of 77.5% [12]. However, the optimal treatment for patients with untreated synchronous oligometastatic NSCLC remains unclear because 70% of the participants in the previously mentioned study were patients with metachronous oligometastatic NSCLC, and over 50% of the participants were previously treated with systemic therapy [12]. Thus, we are conducting a multicentre single-arm phase II study to evaluate the efficacy of LAT at all disease sites following therapy with a combination of pembrolizumab and platinum doublet for patients with untreated synchronous oligometastatic NSCLC.

## Patients and methods

### Participants

Planned enrolment period is 2020 October to 2022 October, and the observation period will be 3-year follow-up period from the time the last patient is enrolled. Key inclusion and exclusion criteria are shown in Table 1. Patients with an activating driver mutation (epidermal growth factor receptor [*EGFR*], anaplastic lymphoma kinase [*ALK*], proto-oncogene 1 [*ROS1*], *BRAF* and *MET*) are not eligible for this study because the initial standard treatment for these population is different. The protocol will be amended to exclude patients with new potential activating driver mutation if approved during the period of this study. Furthermore, multiplex gene tests are not applicable to all patients in clinical practice. Therefore, in some patients, the single-plex gene test will be used to evaluate a few driver mutations such as *EGFR* or *ALK*. Potential participants will be enrolled in this study after receiving a full explanation and obtaining consent of the study.

**Table 1:** Key Inclusion and Exclusion Criteria

**Inclusion criteria**	
1. Age ≥ 20 years, < 75 years	
2. Written informed consent	
3. Histologically or cytologically confirmed NSCLC	
4. Activating driver mutation negative or unknown: epidermal growth factor receptor (*EGFR*), anaplastic lymphoma kinase (*ALK*), proto-oncogene 1 (*ROS1*), *BRAF* and *MET*^a^	
5. No previous chemotherapy	
6. Synchronous oligo-metastatic stage IV disease: maximum of three distant metastases^b^	
7. Suitable candidate for LAT (radiotherapy and/or surgery) to all site of disease, as determined by a multidisciplinary tumour board	
8. Eastern Cooperative Oncology Group (ECOG) performance status score of 0 or 1	
9. No malignant dissemination, pericardial effusion, leptomeningeal metastases, peritoneal dissemination and ascites	
10. Adequate hematologic and organ function	
**Exclusion criteria**	
1. Presence of any other active cancer	
2. Presence of active infections requiring antibiotics	
3. History of active autoimmune disease requiring systemic treatment	
4. History of interstitial lung disease diagnosed or severe chronic obstructive pulmonary disease (COPD)	

^a^*EGFR*/*ALK*/*ROS-1*/*BRAF*/*MET* testing is not mandatory for patients with squamous carcinoma

^b^Based on a previous study, any metastatic thoracic lymph nodes (N1–N3), including in the supraclavicular fossae, were collectively considered a single metastasis

Participating institutions include public hospitals, university hospitals, and cancer centers in Japan. The institutions in the study are; Hokkaido University Hospital, Sendai Kousei Hospita, Niigata Cancer Center Hospital, Juntendo University Hospital, Kanagawa Cardiovascular and Respiratory Center, Nagoya City University Hospital, Aichi Cancer Center Hospital, Aichi Medical University Hospital, Kindai University Hospital, Kansai Medical University Hospital, Kishiwada City Hospital, Osaka City General Hospital, Kobe City Medical Center General Hospital, Kurume University Hospital, Kyushu University Hospital, National Hospital Organization Kyusyu Cancer Center, Kumamoto University Hospital and Shizuoka Caner Center.

### Study design

This study was approved by the central review board of Shizuoka Cancer center (number: 20–1–20-1). The study is registered in the Japan registry of Clinical Trials (jRCTs041200046) and conducted in compliance with the Declaration of Helsinki. Because of the lack of definitive data on efficacy and safety, this study was conducted as an exploratory single-arm phase II trial. Should this study achieve its primary endpoint, we will conduct a phase III trial to evaluate the effect of adding LAT to platinum-doublet chemotherapy plus pembrolizumab for patients with synchronous oligometastatic NSCLC.

**Fig. 1:**
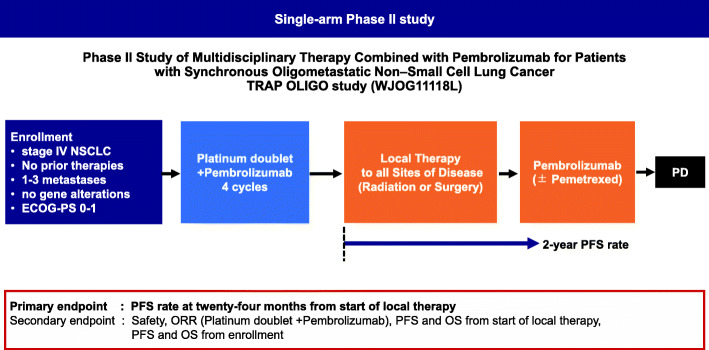
ECOG, Eastern Cooperative Oncology Group; PS, performance status; PD, progressive disease; ORR, objective response rate; PFS, progression-free survival; OS, overall survival

In this single-arm phase II trial, we plan to enrol patients with synchronous oligometastatic NSCLC at diagnosis (defined as ≤3 metastases, according to previous trials and the current trial) [4, 5]. Figure 1 shows the study schema. The Protocol-prescribed treatment will continue until disease progression or met discontinuation criteria for up to 2 years.

All patients will receive 2–4 cycles of systemic treatment as induction therapy. Patients with non-squamous histology will receive pembrolizumab (200 mg/body) + cisplatin (75 mg/m^2^) + pemetrexed (500 mg/m^2^), or pembrolizumab (200 mg/body) + carboplatin (area under the curve [AUC] 5) + pemetrexed (500 mg/m^2^) by intravenous infusion on the first day. Patients with squamous histology will receive pembrolizumab (200 mg/body) + carboplatin (AUC 6) on the first day and nab-paclitaxel (100 mg/m^2^) on days 1, 8, and 15, or pembrolizumab (200 mg/body) + carboplatin (AUC 6) + paclitaxel (200 mg/m^2^) by intravenous infusion on the first day. This treatment, defined as ‘1 cycle’, will be repeated every 3 weeks until 4 cycles.

Patients will receive LAT at all sites of disease within 21–56 days of the last dose of induction therapy. Patients who will receive LAT will be determined by a multidisciplinary tumour board (including medical oncologists, radiation oncologists, and thoracic surgeons). LAT eligibility will be evaluated based on the comorbidity of the patient and the status of the tumour, including the tumour size and location, by a multidisciplinary tumour board. LAT is defined as radiotherapy to all sites of disease or as surgery for thoracic lesions including the primary site, regional lymph nodes, or pulmonary metastases.

Maintenance therapy will start within 42 days of the last day of LAT. Patients with non-squamous histology will receive pembrolizumab (200 mg/body) + pemetrexed (500 mg/m^2^) by intravenous infusion on the first day. Patients with squamous histology will receive pembrolizumab (200 mg/body) by intravenous infusion on the first day.

Tumor assessment will be conducted at baseline, every 6 weeks during the induction therapy, before LAT and every 9 weeks during the maintenance therapy. Tumor response will be evaluated in accordance with the Response Evaluation Criteria in Solid Tumors (RECIST, version 1.1). The prescribed adverse events and any severe adverse events will be recorded on the basis of the National Cancer Institute Common Terminology Criteria for Adverse Events (version 5.0). Concomitant use of anticancer drugs (including molecular targeted drugs and immune checkpoint inhibitors) that may affect protocol treatment is prohibited in this study. There is no special compensation for enrolled patients in this study.

### Data collection and monitoring

All data related to this study is collected through the electric data capturing system throughout enrolment period and follow-up period. The data center of West Japan Oncology Group (WJOG) is supposed to take responsibility for management of data in this study. If the protocol of this study is amended, the WJOG data center will promptly notify the principal investigators at each site. The auditing is planned to conduct in accordance with regulations of WJOG.

The role responsibility of data monitoring committee (DMC) is to review efficacy and safety of this study independently from the investigators. The DMC review reports of severe adverse events from researchers and monitoring reports from the data center. The DMC comprised from four medical doctors who are independent from this study.

### Statistical analysis

The primary endpoint is to evaluate the PFS rate of 24 months from the initiation of LAT. The 24-month PFS rate could be a reliable surrogate marker for the five-year survival rate in locally advanced NSCLC patients [13]. A phase II trial for oligometastatic NSCLC patients (defined by the investigators as 1–3 metastases) who received LAT after standard systemic therapy showed that the median PFS was 11.9 months and the 24-month PFS rate was approximately 25% [5]. Therefore, we set the threshold of the 24-month PFS rate at 25%. In a previous phase II trial of oligometastatic NSCLC patients, the 24-month PFS rate was approximately 50% following treatment with pembrolizumab after LAT, and over half the trial participants were previously treated with systemic therapy [12]. Thus, we expect a 24-month PFS rate of 60% should the trial population increase by a further 10%. Under the current threshold and expected values, 17 patients receiving LAT would be required to achieve 80% power and a lower limit higher than 25% for the confidence interval of PFS rate. In a previous phase II trial, approximately 30% of the enrolled patients did not receive LAT, owing to disease progression during induction systemic therapy [5, 6]. Therefore, 30 patients who start induction therapy will initially be included, considering the possibility of patients dropping out. No interim analysis of this study will be performed.

## Discussion

This phase II study aims to evaluate the efficacy of LAT at all sites of the disease following standard platinum doublet chemotherapy and pembrolizumab therapy in patients with synchronous oligometastatic NSCLC. LAT at all sites of the disease would inherently reduce the overall tumour burden, and this could promote T cell reinvigoration to enhance the efficacy of pembrolizumab [9–11], not just provide local control [2]. The ongoing phase III study might establish LAT at all disease sites following standard platinum doublet chemotherapy as a new standard therapy in patients with oligometastatic NSCLC [14]. This study will provide novel data on the efficacy and safety profile of the combination of LAT and chemotherapy plus immune-checkpoint inhibitors in patients with synchronous oligometastatic NSCLC.

## Conclusion

If the results of this study meet the primary endpoint, we will recommend that the integrated strategy of LAT at all sites of disease following chemotherapy and pembrolizumab for patients with synchronous oligometastatic NSCLC be assessed further in more extensive phase III studies.

## Data Availability

Not applicable.

## Abbreviations

LAT: Local ablative therapy
NSCLC: Non-small cell lung cancer
OS: Overall survival
PD: Progressive disease
PFS: Progression-free survival
PD-1: Programmed cell death 1
